# Persistent sleep symptoms in post-COVID syndrome: a Brazilian prospective clinical study

**DOI:** 10.1055/s-0045-1812890

**Published:** 2025-12-02

**Authors:** Alissa Elen Formiga Moura, Danilo Nunes Oliveira, José Wagner Leonel Tavares Júnior, Alessandra Marangoni Fante, Lívia Leite Góes Gitaí, Paulo Ribeiro Nóbrega, Pedro Braga-Neto, Manoel Alves Sobreira-Neto

**Affiliations:** 1 Universidade Federal do Ceará, Hospital Universitário Walter Cantídio, Departamento de Neurologia, Fortaleza CE, Brazil.; 2 Centro Universitário Christus, Centro de Ciências da Saúde, Fortaleza CE, Brazil.; 3 Universidade Federal do Ceará, Departamento de Medicina Clínica, Sessão de Neurologia, Fortaleza CE, Brazil.; 4 Universidade Federal de Alagoa, Campus A.C. Simões, Maceió AL, Brazil.; 5 Universidade Estadual do Ceará, Centro de Ciências da Saúde, Campus do Itaperi, Fortaleza CE, Brazil.

**Keywords:** COVID-19, Post-Acute COVID-19 Syndrome, Sleep Wake Disorders, Sleep Initiation and Maintenance Disorders, Disorders of Excessive Somnolence

## Abstract

**Background:**

Post coronavirus disease (COVID) syndrome is a frequent condition, resulting from the persistence of symptoms or the development of new ones, after 12 weeks of acute severe acute respiratory syndrome coronavirus 2 infection. The impact on sleep was observed worldwide, whether in the acute phase due to the direct effect of the infection or due to changes in patients' circadian cycle imposed by new routines during quarantine, dysfunctional habits, and social isolation.

**Objective:**

Given the persistence of sleep-related symptoms in patients with long-term COVID syndrome, we decided to follow-up and reassess these patients after 1 year.

**Methods:**

The present is an observational, longitudinal, and prospective clinical study. The study took place in two stages: the first assessment between October 2020 and September 2021, with reassessment after 1 year. Participants underwent the application of the Geriatric Depression Scale (GDS) or the Beck Inventory, Addenbrooke's Cognitive Examination - Revised (ACE-R) and Mini-Mental State Examination (MMSE). The evaluation of sleep disorders involved a comprehensive clinical history obtained through a structured questionnaire.

**Results:**

Of the 46 cases with sleep complaints, 7 refused to return for a new assessment. Thus, 39 patients were included, of which 9 persisted with insomnia (23.07%) and 2 patients persisted with central hypersomnia (5.12%). The persistence of cognitive complaints was also higher in patients with insomnia when compared with those without it ( *N* = 5; 56%, *p* = 0.001).

**Conclusion:**

Given the persistence of patients with insomnia and central hypersomnia, the damage to the central nervous system may be lasting, reinforcing the need for follow-up.

## INTRODUCTION

Coronavirus disease 2019 (COVID-19) is characterized by a clinical disease that varies from asymptomatic infection to fatal disease. 1 Despite efforts to control acute infection, the impacts of COVID-19, in the medium and long term, have become a public health problem. Recent evidence indicates that approximately 80% of people infected with COVID-19 have one or more long-term symptoms. 2 These can be multisystemic and result from symptoms such as fatigue, dyspnea, chest pain, cognitive disorders, headache, anosmia, dysautonomia, insomnia, and other sleep disorders in a condition called long COVID or post-COVID syndrome. 3 4 These symptoms should persist or develop 12 weeks after the acute condition, cannot be better explained by other conditions, and have an impact on the quality and functionality of everyday life. 5

Most sleep studies during the COVID-19 pandemic address insomnia, psychological symptoms, and the impact of disrupted sleep-wake schedules. The presence of insomnia affects around 26 to 45% of patients. 6 Covidsomnia is a new term used to describe sleep disorders experienced during the pandemic, and it encompasses several of these conditions. 7 An impact on sleep has been observed all around the world, either by the direct effect of the infection or by changes in the circadian cycle imposed by new routines started in quarantine, dysfunctional habits, as well as social isolation, anxiety, and depression. The COVID-19 infection can lead to several specific sleep disorder syndromes. 8

In a previous study of our group, we assessed 189 patients with neurological symptoms after COVID-19. Among these patients, 48 (25.3%) had sleep-related complaints. Insomnia was reported by 42 patients (22.2%) and excessive daytime sleepiness by 6 patients (3.17%). Among these last 6 patients, 2 refused to pursue further investigation. 9 Given the persistence of sleep-related symptoms in patients with post-COVID syndrome, we performed, in the present study, the segment and reassessment of these patients after 1 year. The aim of this study is to investigate whether sleep disturbances persist at 1 year of follow-up.

## METHODS

The present is an observational, longitudinal, and prospective clinical study conducted in two stages: the first assessment between October 2020 and September 2021, with reassessment after 1 year.

### Study population

After 1 year of the initial assessment, the 46 patients with sleep disorders were contacted through a telephone interview to check the persistence of symptoms and new related complaints, and they were invited to participate in a new face-to-face medical assessment of clinical, cognitive, and sleep-related aspects. A protocol similar to the one used in the initial assessment was used (neurological and related to sleep disorders). 9 All assessments were performed by the same examiner, a specialist in neurology and sleep medicine.

Patient data were collected using a semi-structured questionnaire. We assessed sociodemographic, COVID infection severity (hypoxemia, systemic involvement, and/or need for hospital admission), neurological symptoms, cognitive performance, and psychiatric symptoms.

Psychiatric disorders were evaluated through a structured interview conducted by a neurologist. Participants underwent the application of the Geriatric Depression Scale (GDS) to assess mood, or the Beck Inventory, depending on age. We used a cutoff point of 3 on the GDS and 10 on the Beck inventory for the diagnosis of depression. 7 The disorders were classified according to the Diagnostic and Statistical Manual of Mental Disorders (DSM-5). Participants were submitted to the Addenbrooke's Cognitive Examination–Revised (ACE-R) and Mini-Mental State Examination (MMSE). Cut-off values of 58, 76, and 83 points were used for patients with < 4, 4 to 8, and > 8 years of education, respectively, in the ACE-R. 10

For the MMSE, cutoffs of 19 for illiterate patients and 24 for literate patients were used. 11 12 Cognitive diagnosis was established according to scores on the MMSE and ACE-R. Dementia was diagnosed when patients presented cognitive impairment with associated functional impairment, while MCI and DCS were defined in the absence of functional impairment and, respectively, with objectively determined cognitive impairment. 13

### Sleep disorders assessment

The evaluation of sleep disorders involved a comprehensive clinical history obtained through a structured questionnaire designed to capture the most common diagnoses in sleep medicine. Additionally, the assessment included the Epworth Sleepiness Scale and a sleep diary. Further diagnostic testing was performed only in patients presenting with excessive daytime sleepiness (EDS), and included overnight polysomnography (PSG), the Multiple Sleep Latency Test (MSLT), and actigraphy, all conducted according to the American Association of Sleep Medicine (AAMS) protocols. 14 Sleep disorder diagnoses were established in accordance with the 2014 International Classification of Sleep Disorders, Third Edition (ICSD-3), from the AAMS. 14

### Statistical analysis

Categorical variables were described as absolute frequencies and percentages, while numerical variables were described as averages and standard deviations (SDs).

The Kolmogorov–Smirnov test was used to assess the distribution of the variables. For those with a normal distribution, parametric tests such as the Student's *t* -test or analysis of variance (ANOVA) were applied. Variables that did not follow a normal distribution were analyzed with nonparametric tests, including the Mann-Whitney U and Kruskal-Wallis tests. For the correlation analysis of numerical variables, Pearson's correlation coefficient was used for normally distributed variables, while Spearman's rank was used for non-normally distributed ones. Categorical variables were compared across two or more groups using the chi-squared or Fisher's exact tests, depending on the expected frequency in the contingency table cells.

Two-tailed *p* -values < 0.05 were considered statistically significant. Statistical analyses were performed with jamovi (the jamovi project), version 1.0.

### Ethical aspects

The study project was approved by the Research Ethics Committee of the Hospital Universitário Walter Cantídio under number 4,092,933 and complies with the ethical principles of the Declaration of Helsinki. The free and informed consent form was applied. The document was read and agreed upon by the patients or legal representatives, with registration, before beginning the protocol, ensuring respect for patients' freedom to refuse to participate or to withdraw their consent at any stage of the research, without penalty or prejudice to their care, with, in addition, a guarantee of secrecy to ensure their privacy regarding the research. All patients signed the form, with the right to secrecy and confidentiality of the information obtained and the freedom to refuse participating in the proposed activities and questions.

## RESULTS

Of the 46 patients contacted, 7 refused to return for a new evaluation. Therefore, 39 patients were included, among whom 9 persisted with insomnia (23.07%). Initial insomnia was present in 6 patients (66,6%), followed by maintenance insomnia in 3 (33,33%), and 2 who persisted with central hypersomnia (5.12%).

### Clinical reassessment

In the 2 ^nd^ stage of reassessments, from 4 patients followed up with the diagnosis of central hypersomnia, 2 had persisting symptoms (50%), requiring maintenance of treatment with wakefulness-promoting agents. They also persisted with clinical complaints related to memory and attention, only without functional impairment in the applied cognitive tests. Also, patients presented with depressive and anxiety symptoms, with satisfactory control through the use of medication. The other characteristics are described in Table 1 .

**Table 1:** Clinical data of patients who persisted with central hypersomnia after COVID-19 infection

Variables	Case one	Case two
Sex	F	M
Age (years)	35	42
Race	Brown	Caucasian
Time after COVID-19 infection (months)	26	28
Need for hospitalization	N	N
Cognitive impairment	Y	Y
Mood disorder	Y	Y
Start time of CH (months)	22	23
Alcoholism	N	N
Smoking	N	N
Comorbidities	Depression, endometriosis	N
Current Epworth Sleepiness Scale	6	7
MMSE	30	28
ACE-R	93	83
Snoring	N	N
Fatigue	N	N
Restorative sleep	N	N
Cataplexy	N	N
RLS symptoms	N	N
Treatment	Methylphenidate 20 mg/d	Lisdexamfetamine 70 mg/d

Abbreviations: ACE-R, Addenbrooke's Cognitive Examination–Revised; CH, central hypersomnia; COVID-19, coronavirus disease 2019; MMSE, Mini Mental State Examination; N, no; RLS, restless leg syndrome; Y, yes.

Table 2 describes the clinical data of the reevaluation of patients after 1 year of follow-up. A significant frequency of patients still had insomnia at the 1-year follow-up ( *N* = 9; 23.07%). Multivariate analysis showed persistent cognitive complaints were also significantly higher in patients with insomnia when compared with those without (n = 5; 56%; *p* = 0.001).

**Table 2:** Clinical data of reassessed patients with insomnia

Variables		No persistent insomnia (n = 28)	With persistent insomnia (n = 9)	*p-* value
Age, years		51 ± 11)	48 ± 12)	0.472
Male, n (%)		7 (25%)	5 (56%)	0.262
Education (schooling time)	Illiterate	0 (0%)	1 (11%)	0.099
	>12	16 (57%)	8 (89%)	
Time between COVID infection and last assessment, months		23.3 ± 6	24.7 ± 5.7	0.671
Alcoholism, n (%)		8 (29%)	3 (33%)	0.744
Smoking, n (%)		3 (11%)	2 (22%)	0.577
Hospitalization due to COVID, n (%)		7 (25%)	1 (9.1%)	0.649
Cognitive decline during first assessment, n (%)		23 (82%)	6 (67%)	0.513
Persistence of cognitive decline at second assessment, n (%)		11 (38.1%)	5 (56%)	0.001
MMSE		28,78 (1.18)	28.29 (1.12)	0.265
ACE-R		87.11 (6.02)	84.79 (9.39)	0.480
Diagnosis of dementia, n (%)		0	0	–
Diagnosis of depression, n (%)		6 (21%)	3 (33%)	0.657
Diagnosis of anxiety disorder, n (%)		6 (21%)	56 (56%)	0.091
APOE genotype	E2/E3	1 (4.5)	0	0.762
	E3/E3	13 (59%)	6 (75%)	
	E3/E4	8 (36%)	2(25%)	

Abbreviations: ACE-R, Addenbrooke's Cognitive Examination–Revised; APOE, apolipoprotein E; COVID, coronavirus disease; MMSE, Mini Mental State Examination.

Source: Elaborated by the author with research data.

## DISCUSSION

The present study demonstrated that a group of patients have persistent sleep complaints in a cohort of patients with post-COVID syndrome. Persisting long COVID symptoms in some patients were observed after 24 months of acute infection. As far as we know, this is the cohort study with the longest duration of follow-up, which evaluated the frequency and the evolution of sleep disorders in adults after COVID-19 infection.

One explanation for the persistence of sleep complaints in COVID-19 patients could be dysfunction of brainstem nuclei. 11 This may be justified by the high presence in the brainstem of angiotensin converting enzyme type 2 receptors and possibly neuropilin-1. These receptors are used by severe acute respiratory syndrome coronavirus 2 (SARS-CoV-2) to infect cells and promote cellular damage and dysfunction. 12 15 16 There are cell nuclei in the brain stem involved with the regulation of the sleep–wake cycle that could be affected by the pathological process of COVID-19, such as periaqueductal gray matter, dorsal raphe nucleus, pedunculopontine nucleus, and laterodorsal tegmental nucleus, among others. 15 16

A greater persistence of cognitive complaints was found in the group of patients with insomnia, even ruling out possibly misleading factors, such as hospitalization, depression, and anxiety. The damage to the central nervous system (CNS) may be long-lasting, with a cognitive case characterized by impairments in attention, concentration, working memory, and processing speed, subtle changes may go unnoticed in habitual cognitive screening assessments. 17 Moreover, the coexistence of these symptoms may indicate an important direction in identifying clinical subtypes of post-COVID syndrome for future studies, as it can produce a wide range of neurological symptoms. 17 As this syndrome still has no treatment, better clinically defining this group of patients can assist in the definition of biomarkers and subsequent better recruitment for clinical trials. 18 19

Regarding the pathophysiology responsible for cognitive symptoms in long COVID, cerebrovascular dysfunction, endothelial injury, and neuroinflammation take importance. 20 21 22 23 It also results from an activation of the inflammasome with subsequent dysregulated inflammatory response and persistence of proinflammatory activity after the acute phase of the disease.

In fact, structural and functional changes have been demonstrated in the brains of patients with long COVID and cognitive symptoms. 24 25 26 Among these changes, in a longitudinal study, Douaud et al. used magnetic resonance imaging (MRI) of the skull to demonstrate gray matter atrophy in the orbitofrontal cortex and parahippocampal gyrus of patients with long COVID. 24 Additionally, Teller et al. used Diffusion Tensor Imaging to show a difference in restriction to frontal diffusion (smaller) compared with cerebellum (larger). 25 Kim et al. used arterial spin labeling and demonstrated hypometabolism in nuclei of the base, thalamus, and orbitofrontal cortex comparing patients with COVID-19, including long COVID, with controls. 26

Even in patients with mild systemic cases, we could observe cognitive complaints. Neuroinflammation can cause dysregulation of glial and neuronal cells and, ultimately, neural circuit dysfunction, which negatively affects cognitive and neuropsychiatric functions. 27 Additionally, neuronal cell dysregulation can be immune-mediated. These changes can lead to activation of microglia in the subcortical and hippocampal white matter areas. 27

Microglia are macrophage cells residing in the CNS. Although they contribute to the system's homeostasis and the refinement of neuronal networks by removing dendritic spines and synapses during the development of neurons, microglia can make the transition to an activated neurotoxic state. In the subcortical white matter, microglial activation was associated with loss of precursor and mature oligodendrocytes; consistently, there was also loss of myelin and myelinated axons for at least 7 weeks after the start of infection. The loss of myelinated axons impairs the structure and function of neuronal networks. 28 In the hippocampus, microglia activation was associated with inhibited neurogenesis, which could explain impaired memory formation in patients. 28 Understanding the mechanisms involved in this new condition allows us to assess a commonality in long COVID, as well as targeted therapeutic possibilities.

The main cognitive profile found in our study, through screening tests, was of patients with subjective cognitive decline. The literature frequently describes the critical role of sleep in maintaining cognitive health, noting that this relationship is bidirectional. For instance, patients with subjective cognitive decline experience sleep disorders at a higher frequency relative to controls. 29

Conversely, animal studies indicate that prolonged sleep restriction or interruption has cumulative effects on the brain, for example, leading to reduced proliferation of hippocampal cells and widespread changes in the microstructure of white matter. Meanwhile, in humans, experimental studies have shown that sleep deprivation is associated with significantly reduced performance on tests of attention, labor, and short-term memory. 30 Thus, the relationship between cognitive symptoms and insomnia in long COVID patients deserves future studies of cause and effect.

Recent studies suggest that the deposition of β-amyloid protein in the brain will be in the basal forebrain, a structure involved in sleep regulation and implicated in the progression of Alzheimer's disease. 30 31 Alternatively, these initial disruptions in sleep architecture may be the impetus for the development of the disease, generating disruptions in the glymphatic clearance of waste, which would lead to further accumulation of β-amyloid plaques and tau tangles. 31 32 33

There was no evidence in our sample of an association between patients who persisted with sleep disorders and psychiatric symptoms, such as depression and anxiety. Baril et al. showed that more severe insomnia symptoms were associated with lower performance on global cognition, which was especially apparent in the apolipoprotein E (APOE) ε4 allele carriers, suggesting that poor sleep might be particularly detrimental when the brain is already vulnerable to neurodegeneration. 34 The presence of apolipoprotein E2 to E4 also showed no difference between patients who persisted with insomnia. The reduced number of participants may have impaired these assessments.

In our initial sample, we described 3 patients proven to have hypersomnia of central origin after COVID-19 infection, one of whom met the criteria for narcolepsy. 9 It is noteworthy that these young individuals who did not have symptoms before COVID infection, strongly suggesting an association with the disease. After 24 months of acute infection, two patients had persistent symptoms, controlled with the use of wakefulness-promoting medications, which may lead to hypothalamic damage. 9

The high prevalence of ACE2 receptors in the CNS favors a tropism of COVID-19 action, which could result in encephalitis (either by direct or immune-mediated action of the virus), which would result in excessive daytime sleepiness (EDS) due to lesions in the hypothalamus, temporal lobes, hippocampus, and brainstem. 20 21 35 36 37 As potential neuroimmunological diseases, hypersomnias (narcolepsy, Kleine-Levin syndrome [KLS], and idiopathic hypersomnia) can be triggered by external factors such as upper respiratory tract infection, H1N1 influenza, or neuroimmunological response to vaccination. 38 39

Similar to other neurotropic viruses, the COVID-19 virus can cause CNS encephalitis, as well as hypothalamic impairment. The literature presents one case of KLS relapse in a young patient, without other precipitating factors, after COVID-19 infection. 40 This reinforces the findings of central hypersomnia found in our sample. 9

This study had some limitations, such as the small sample size, limiting the analysis and generalizability of the findings, the absence of a control group prevents direct comparisons, and the absence of PSG, MSLT, and actigraphic investigation in all patients. On the other hand, only a few patients declined to participate in the reevaluation, resulting in a rate of 85%. An accurate clinical assessment of sleep symptoms in post-COVID patients was conducted after a significant time interval and was correlated with a concurrently cognitive evaluation.

In conclusion, sleep disorders are a common persistent symptom in patients with post-COVID syndrome, indicating a possible long-lasting CNS damage. Besides, patients who persisted with insomnia were significantly associated with persistent cognitive complaints, reinforcing the need for segment of this cohort.

## References

[1] China Novel Coronavirus Investigating and Research Team Zhu N Zhang D Wang W A Novel Coronavirus from Patients with Pneumonia in China, 2019 N Engl J Med 2020 382 08 727 733 10.1056/nejmoa2001017 31978945 PMC7092803

[2] Alkodaymi M S Omrani O A Ashraf N Prevalence of post-acute COVID-19 syndrome symptoms at different follow-up periods: a systematic review and meta-analysis Clin Microbiol Infect 2022 28 05 657 666 10.1016/j.cmi.2022.01.014 35124265 PMC8812092

[3] Chen Y Xu Z Wang P New-onset autoimmune phenomena post-COVID-19 vaccination Immunology 2022 165 04 386 401 10.1111/imm.13443 34957554

[4] Koczulla A R Ankermann T Behrends U [S1 Guideline Post-COVID/Long-COVID] Pneumologie 2021 75 11 869 900 10.1055/a-1551-9734 34474488

[5] Nalbandian A Sehgal K Gupta A Post-acute COVID-19 syndrome Nat Med 2021 27 04 601 615 10.1038/s41591-021-01283-z 33753937 PMC8893149

[6] Jacobs L G Paleoudis E G Lesky-Di Bari D Persistence of symptoms and quality of life at 35 days after hospitalization for COVID-19 infection PLoS One 2020 15 12 e0243882 10.1371/journal.pone.0243882 33306721 PMC7732078

[7] Gupta R Pandi-Perumal S R COVID-Somnia: How the Pandemic Affects Sleep/Wake Regulation and How to Deal with it? Sleep Vigil 2020 4 02 51 53 10.1007/s41782-020-00118-0 33289005 PMC7711056

[8] Coelho F MS Czuma R Ticotsky A Maley J Mullington J M Thomas R J Sleep disorder syndromes of post-acute sequelae of SARS-CoV-2 (PASC) / Long Covid Sleep Med 2024 123 37 41 10.1016/j.sleep.2024.08.030 39236463

[9] Moura A EF Oliveira D N Torres D M Central hypersomnia and chronic insomnia: expanding the spectrum of sleep disorders in long COVID syndrome - a prospective cohort study BMC Neurol 2022 22 01 417 10.1186/s12883-022-02940-7 36352367 PMC9643986

[10] Carvalho V A Caramelli P Brazilian adaptation of the Addenbrooke's Cognitive Examination-Revised (ACE-R) Dement Neuropsychol 2007 1 02 212 216 10.1590/s1980-57642008dn10200015 29213390 PMC5619571

[11] Casez O Willaume G Grand S Teaching NeuroImages: SARS-CoV-2-Related Encephalitis: MRI Pattern of Olfactory Tract Involvement Neurology 2021 96 04 e645 e646 10.1212/wnl.0000000000011150 33154083

[12] Shimizu S Shimizu T Nakamura K Higashi Y Saito M Angiotensin II, a stress-related neuropeptide in the CNS, facilitates micturition reflex in rats Br J Pharmacol 2018 175 18 3727 3737 10.1111/bph.14439 29981238 PMC6109218

[13] Elahi F M Miller B L A clinicopathological approach to the diagnosis of dementia Nat Rev Neurol 2017 13 08 457 476 10.1038/nrneurol.2017.96 28708131 PMC5771416

[14] American Academy of Sleep Medicine International classification of sleep disorders – third edition (ICSD-3) AASM Resource Library 2014 281 09 287 292

[15] Fleming R Grattan R Bohmova K Case of COVID-19 in a 5-week-old baby BMJ Case Rep 2020 13 09 e236330 10.1136/bcr-2020-236330 PMC7470488 32878835

[16] Young C N Davisson R L Angiotensin-II, the Brain, and Hypertension: An Update Hypertension 2015 66 05 920 926 10.1161/hypertensionaha.115.03624 26324508 PMC4600031

[17] Gill C Cho T A Neurologic Complications of COVID-19 Continuum (Minneap Minn) 2023 29 03 946 965 10.1212/con.0000000000001272 37341337

[18] Quan M Wang X Gong M Wang Q Li Y Jia J Post-COVID cognitive dysfunction: current status and research recommendations for high risk population Lancet Reg Health West Pac 2023 38 100836 10.1016/j.lanwpc.2023.100836 37457901 PMC10344681

[19] Bonilla H Peluso M J Rodgers K Therapeutic trials for long COVID-19: A call to action from the interventions taskforce of the RECOVER initiative Front Immunol 2023 14 1.129459E6 10.3389/fimmu.2023.1129459 PMC10034329 36969241

[20] Herrera E Jr Caramelli P Silveira A SB Nitrini R Epidemiologic survey of dementia in a community-dwelling Brazilian population Alzheimer Dis Assoc Disord 2002 16 02 103 108 10.1097/00002093-200204000-00007 12040305

[21] Desforges M Le Coupanec A Stodola J K Meessen-Pinard M Talbot P J Human coronaviruses: viral and cellular factors involved in neuroinvasiveness and neuropathogenesis Virus Res 2014 194 145 158 10.1016/j.virusres.2014.09.011 25281913 PMC7114389

[22] Muccioli L Pensato U Cani I Guarino M Cortelli P Bisulli F COVID-19-Associated Encephalopathy and Cytokine-Mediated Neuroinflammation Ann Neurol 2020 88 04 860 861 10.1002/ana.25855 32715524

[23] Nordvig A S Fong K T Willey J Z Potential Neurologic Manifestations of COVID-19 Neurol Clin Pract 2021 11 02 e135 e146 10.1212/cpj.0000000000000897 33842082 PMC8032406

[24] Douaud G Lee S Alfaro-Almagro F SARS-CoV-2 is associated with changes in brain structure in UK Biobank Nature 2022 604(7907):697 707 10.1038/s41586-022-04569-5 35255491 PMC9046077

[25] Teller N Chad J A Wong A Feasibility of diffusion-tensor and correlated diffusion imaging for studying white-matter microstructural abnormalities: Application in COVID-19 Hum Brain Mapp 2023 44 10 3998 4010 10.1002/hbm.26322 37162380 PMC10258529

[26] Kim W SH Ji X Roudaia E MRI Assessment of Cerebral Blood Flow in Nonhospitalized Adults Who Self-Isolated Due to COVID-19 J Magn Reson Imaging 2023 58 02 593 602 10.1002/jmri.28555 36472248 PMC9877942

[27] Monje M Iwasaki A The neurobiology of long COVID Neuron 2022 110 21 3484 3496 10.1016/j.neuron.2022.10.006 36288726 PMC9537254

[28] Fernández-Castañeda A Lu P Geraghty A C Mild respiratory COVID can cause multi-lineage neural cell and myelin dysregulation Cell 2022 185 14 2452 2.468E19 10.1016/j.cell.2022.06.008 35768006 PMC9189143

[29] Pace-Schott E F Spencer R MC Sleep-dependent memory consolidation in healthy aging and mild cognitive impairment Curr Top Behav Neurosci 2015 25 307 330 10.1007/7854_2014_300 24652608

[30] Lim A SP Yu L Kowgier M Schneider J A Buchman A S Bennett D A Modification of the relationship of the apolipoprotein E ε4 allele to the risk of Alzheimer disease and neurofibrillary tangle density by sleep JAMA Neurol 2013 70 12 1544 1551 10.1001/jamaneurol.2013.4215 24145819 PMC3859706

[31] Wennberg A MV Wu M N Rosenberg P B Spira A P Sleep Disturbance, Cognitive Decline, and Dementia: A Review Semin Neurol 2017 37 04 395 406 10.1055/s-0037-1604351 28837986 PMC5910033

[32] Kadotani H Kadotani T Young T Association between apolipoprotein E epsilon4 and sleep-disordered breathing in adults JAMA 2001 285 22 2888 2890 10.1001/jama.285.22.2888 11401610

[33] Lim J Dinges D F A meta-analysis of the impact of short-term sleep deprivation on cognitive variables Psychol Bull 2010 136 03 375 389 10.1037/a0018883 20438143 PMC3290659

[34] Baril A A Beiser A S Sanchez E Insomnia symptom severity and cognitive performance: Moderating role of APOE genotype Alzheimers Dement 2022 18 03 408 421 10.1002/alz.12405 34310026 PMC8802306

[35] Evelina PIMS-TS Study Group Sa M Mirza L Carter M Systemic Inflammation Is Associated With Neurologic Involvement in Pediatric Inflammatory Multisystem Syndrome Associated With SARS-CoV-2 Neurol Neuroimmunol Neuroinflamm 2021 8 04 e999 10.1212/nxi.0000000000000999 33850037 PMC8054962

[36] Marques L M Marques S R Costa O Freitas E Machado Á COVID-19-Associated Encephalitis: Two Case Reports Cureus 2022 14 03 e23243 10.7759/cureus.23243 35342667 PMC8927857

[37] Marčić M Marčić L Marčić B SARS-CoV-2 Infection Causes Relapse of Kleine-Levin Syndrome: Case Report and Review of Literature Neurol Int 2021 13 03 328 334 10.3390/neurolint13030033 34294673 PMC8299328

[38] Mignot E Black S Narcolepsy risk and COVID-19 J Clin Sleep Med 2020 16 10 1831 1833 10.5664/jcsm.8668 32621581 PMC7954017

[39] Montplaisir J Petit D Quinn M J Risk of narcolepsy associated with inactivated adjuvanted (AS03) A/H1N1 (2009) pandemic influenza vaccine in Quebec PLoS One 2014 9 09 e108489 10.1371/journal.pone.0108489 25264897 PMC4180737

[40] Nasrullah A Javed A Ashraf O Malik K Possible role of COVID-19 in the relapse of Klein-Levin Syndrome Respir Med Case Rep 2021 33 101445 10.1016/j.rmcr.2021.101445 34094848 PMC8164508

